# Sustainability of physical exam skills in a resident-led curriculum in a large internal medicine program with competency based medical education

**Published:** 2018-11-12

**Authors:** Don Thiwanka Wijeratne, Siddhartha Srivastava, Barry Chan, Wilma Hopman, Benjamin Thomson

**Affiliations:** 1 Division of Internal Medicine, Faculty of Medicine, Queen’s University, Ontario, Canada; 2 Division of Nephrology, Faculty of Medicine, Queen’s University, Ontario, Canada

## Abstract

**Background:**

Competency Based Medical Education (CBME) designates physical examination competency as an Entrustable Professional Activity (EPA). Considerable concern persists regarding the increased time burden CBME may place on educators. We developed a novel physical examination curriculum that shifted the burden of physical examination case preparation and performance assessment from faculty to residents. Our first objective was to determine if participation led to sustainable improvements in physical examination skills. The second objective was to determine if resident peer assessment was comparable to faculty assessment.

**Methods:**

We selected physical exam case topics based on the Objectives of Training in the Specialty of Internal Medicine as prescribed by the Royal College of Physicians and Surgeons of Canada. Internal Medicine residents compiled evidence-based physical exam checklists that faculty reviewed before distribution to all learners. Physical exam practice sessions with whole-group demonstration followed by small-group practice sessions were performed weekly. We evaluated this pilot curriculum with a formative OSCE, during which a resident peer and a faculty member simultaneously observed and assessed examinee performance by.

**Results:**

Participation in the novel curriculum practice sessions improved OSCE performance (faculty score mean 78.96 vs. 62.50, p<0.05). Peer assessment overestimated faculty scores (76.2 vs. 65.7, p<0.001), but peer and faculty assessments were highly correlated (R^2^ = 0.73 (95% CI 0.50-0.87).

**Conclusion:**

This novel physical examination curriculum leads to sustainable improvement of physical examination skills. Peer assessment correlated well with the gold standard faculty assessment. This resident-led physical examination curriculum enhanced physical examination skills in a CBME environment, with minimal time commitment from faculty members.

## Introduction

With the advent of Competency Based Medical Education (CBME), physical examination is a core skill designated as a consistent milestone of many Entrustable Professional Activities (EPA).^1^ However, since the 1960s, physical examination proficiency amongst trainees continues to remain below expectation,^2-7^ and its importance and emphasis have waned over the decades.^8^ Numerous educational interventions have shown variable success, including structured curriculum,^8^ multimedia-assisted teaching,^9^ simulation training,^10^ feedback,^11^ and instructor variation.^12-14^

The Objectives of Training in Specialty of Internal Medicine of the Royal College of Physicians and Surgeons of Canada (RCPSC) include that trainees should be able to perform “a focused physical examination that is relevant and accurate for… diagnosis and/or management.” However, there is no standardized curriculum defining the breadth of scope and depth of knowledge.^15^ Thus, trainees continue to have varying expectations for an ill-defined standard. Furthermore, creation of a new physical examination curriculum to be concordant with CBME has the practical challenge of being sustainable given limitations in manpower, organization, finances, and faculty time. Faculty remain concerned that CBME demands greater time investment, when they have other competing priorities.^16-18^ Thus, how to teach physical examination skills effectively in a CBME environment, in a way that minimizes time commitments for faculty educators, remains uncertain yet a pressing concern.

The authors constructed, implemented, and evaluated a pilot, two-phased, structured, resident-led physical examination focused curriculum for the Core Internal Medicine and the General Internal Medicine Fellowship trainees (PGY 1-4) to address CBME requirements. The curriculum was dependent on resident learners, with minimal faculty involvement.

The first objective of our study was to determine if participation in the pilot curriculum led to sustainable improvements in physical examination skills measured by performance on a formative OSCE examination. The secondary objective was to determine if peer assessments were comparable to faculty assessments, determined by simultaneous peer and faculty assessments in a formative OSCE.

## Methods

### Setting

We developed a physical examination curriculum for the Internal Medicine Training Program (PGY 1-4) of Queen’s University (Kingston, Canada) which consists of 67 residents.

### Development of physical examination curriculum

We selected physical exam topics based on the Objectives of Training in the specialty of Internal Medicine as prescribed by the RCPSC. Internal Medicine residents in their second or third post-graduate year volunteered to compile evidence-based physical exam checklists from a number of recommended physical exam references including The Rational Clinical Examination: Evidence-based clinical diagnosis,^19^ Evidence-Based Physical Diagnosis,^20^ and Clinical Examination: A Systematic Guide to Physical Diagnosis.^21^ They organized checklists into general inspection, then system-based examination (e.g., Cardiac, Neurological, Abdominal, etc.), and finally evidence-based special tests. Special tests were not considered part of the standard system-based physical examination. We described special tests in the checklist document, with references provided. Checklists were evaluated, refined and finalized by Internal Medicine faculty prior to distribution to all learners (Appendix A).

### Implementation of physical examination curriculum

Practice physical examination sessions were incorporated into weekly academic half-day sessions. A single physical examination checklist for a specific physical exam topic (e.g., physical exam for Myasthenia Gravis) was distributed to all learners at least two days prior to the learning activity, with paper copies provided during the activity. These physical exam practice sessions were conducted first as a large group demonstration. This was facilitated by one to two faculty members who were Fellows of RCPSC in Internal Medicine. The faculty facilitator introduced a clinical scenario pertinent to the topic, then the resident checklist creator demonstrated the physical exam scenario with a resident peer as the standardized patient. Follow-up questions were then discussed with the group, and feedback was provided on the performance by the faculty members. The checklist was also reviewed after the physical exam scenario was performed, to assure all physical exam maneuvers were demonstrated correctly. This was followed by small-group breakout practice sessions, subdivided to one of eight available examination rooms, that were facilitated by faculty. Twenty residents contributed one topic each, so twenty topics were taught using this pilot physical examination curriculum, from July 2016 to February 2017.

### Assessment of physical examination curriculum

In March 2017, a voluntary formative Objective Structured Clinical Examination (OSCE) was organized, to evaluate physical examination performance on four of the twenty topics that had been taught and practiced during the pilot curriculum. There were 36 residents who participated in the voluntary formative OSCE. Scoring sheets for these stations aligned with the checklists developed for the practice sessions with follow-up questions added to each station to assess critical thinking and knowledge application. This checklist and follow-up questions were used to calculate a raw score. A 10-point rubric was used to assign a global, general impression score, with the highest score being 10 and the lowest score being 0 (Appendix B). The raw and global scores were converted to percentages and added to determine combined scores.

Two residents were paired to complete the examination circuit, consisting of four stations. The two residents alternated between being an examiner, marking physical examination performance on the checklist, or the examinee who performed the physical exam upon the resident examiner. No standardized patients were used in any of the OSCE stations. Specific instructions were provided at each station, with two minutes to read the instructions and stem, 10 minutes for the station, and three minutes for feedback. Three identical circuits of four OSCE stations ran simultaneously. In one of the circuits, we performed faculty assessment at each station, by observing through a one-way window that had an unimpeded view of the entire room, and listening via headphones to all sounds produced within the same room. Faculty members at each examination station used the same scoring sheet as resident peer examiners; and there was no communication between faculty and resident peer examiners.

Residents who created checklists were permitted to participate in the formative OSCE. However, if a resident had previously created the checklist for an OSCE physical exam station, he or she was to be the examiner, but not to be examined in such a scenario.

### Data Analysis- Objective 1

Multivariable regression models were used to determine if participation in the novel curriculum physical exam practice sessions improved examinee performance on the formative OSCE, after adjusting for the clinical scenario and PGY level of the examinee. Performance on the formative OSCE was defined as the peer raw score (regression model 1) or the faculty raw score (regression model 2).

### Data Analysis- Objective 2

Raw, global and combined scores were calculated as percentages. Peer and faculty scores were reported as means and standard deviations (SD) by examinee/examinee PGY level and the station topic. As the scores were normally distributed groups’ scores were compared using paired t-test. Statistical significance was set at p<0.05. The 95% confidence intervals of these comparisons were derived. The correlation between peer and faculty scores was determined using Pearson’s Correlation Coefficients.

### Ethics

Ethics approval was obtained through Queen’s University Health Sciences Research Ethics Board, Identification number 6022756. Informed consent was waived as per the Research Ethics Board approval.

## Results

### Participants

There were 72 encounters in the formative OSCE assessment. Faculty observed and evaluated 38.9% encounters (28/72). Examinee participation in the facilitated practice physical exam sessions that corresponded to the OSCE stations was 26.4% (19/72) (Table 1).

**Table 1:** Formative OSCE characteristics

Formative OSCE Encounter Type	N (%)
Total	72 (100%)
Faculty Observed	28 (38.9%)
Examinee participated in Matched Pilot Curriculum Practice Session	19 (26.4%)
Examinee Post Graduate Training Year: 1	22 (30.5%)
2	16 (22.2%)
3	18 (25.0%)
4	16 (22.2%)

### Objective 1: Sustainability of Physical Examination Skills

Participation in the curriculum practice physical examination sessions were associated with higher faculty raw (79.0 vs 62.5, p<0.05) and combined (75.3 vs 61.8, p<0.05) scores (Table 2).

**Table 2:** Effect of pilot curriculum physical examination practice on formative OSCE performance

	Prior Exposure		
	Yes	No	P
**Peer Assessment**			
Number	19	53	
Raw Score	81.4	75.2	0.16
Global Score	78.5	82.6	0.17
Combined Score	76.9	81.9	0.12
**Faculty Assessment**			
Number	8	20	
Raw Score	**79.0**	**62.5**	**<0.05**
Global Score	72.5	61.0	0.08
Combined Score	**75.3**	**61.8**	**<0.05**

* Bolded numbers are statistically significant to p<0.05

In a multivariate model adjusted for the clinical scenario and PGY level of the examinee, residents who participated in novel curriculum practice sessions showed a non-statistically significant trend to improvement in peer raw score (p=0.06, difference = + 6.5 points). Resident participation in novel curriculum practice sessions were associated with higher faculty raw scores on the formative OSCE in another multivariate model with similar adjustments (p=0.01, difference = +19.8 points) (Table 3).

**Table 3:** Multivariate regression model data for peer raw (Model 1) and faculty raw (Model 2) scores

			95% Confidence Interval	
	Beta	P	Lower Limit	Upper Limit
**MODEL 1 (Peer Raw Scores)**				
Constant	62.31	0.00	54.50	70.11
SCENARIO: Meningitis	18.06	0.00	9.74	26.38
Splenomegaly	28.17	0.00	19.86	36.49
Osteoporosis	16.29	0.00	8.05	24.52
POST-GRADUATE YEAR^**^: 1	-0.23	0.96	-8.54	8.08
2	-4.35	0.33	-13.15	4.44
3	-7.13	0.11	-15.80	1.54
Examinee Participation in Pilot Curriculum Practice Session	6.50	0.06	-0.28	13.28
				
**MODEL 2 (Faculty Raw Scores)**				
Constant	51.22	0.00	34.60	67.85
SCENARIO: Meningitis	17.68	0.05	-0.02	35.37
Splenomegaly	11.31	0.19	-6.17	28.79
Osteoporosis	-3.33	0.69	-20.60	13.94
POST-GRADUATE YEAR^***^: 1	8.95	0.32	-9.14	27.04
2	4.66	0.49	-9.27	18.59
**Examinee Participation in Pilot Curriculum Practice Session**	**19.79**	**<0.01**	**4.98**	**34.60**

* Reference for Scenario (Models 1 and 2) is Osteoarthritis versus Rheumatoid Arthritis

** Reference for Model 1 Post-Graduate Year is Post-Graduate Year 4

*** Reference for Model 2 Post-Graduate Year is Post-Graduate Year 3

### Objective 2: Peer versus Faculty Assessments

**Figure 1:**
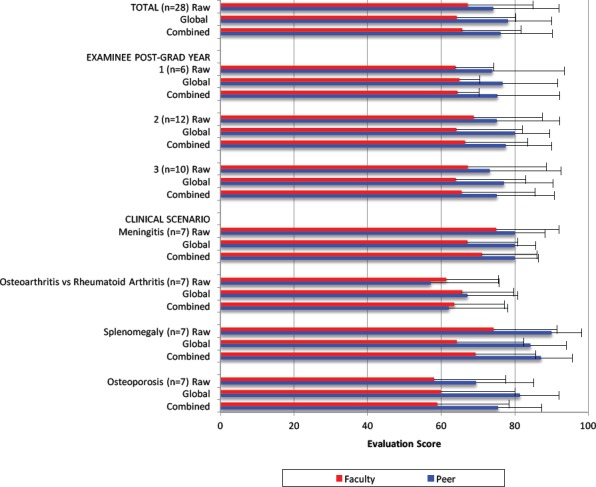
Peer and faculty assessment scores for consanguineous physical exam stations on formative OSCE

In the formative OSCE exam, peer assessment scores exceeded faculty assessment scores for raw (74.2 vs. 67.2, p<0.01), global (78.2 vs 64.3, p<0.001) and combined (76.2 vs 65.7, p<0.001) scores (Table 1). Peer assessment scores were higher than faculty assessment scores for post-graduate year 2 raw (75.1 vs 68.9, p<0.05), global (80.0 vs 64.2, p<0.05) and combined (77.6 vs 66.5, p<0.05) scores, and for post-graduate year 3 global scores (77.0 vs 64.0, p<0.01). Peer assessment scores were also higher than faculty assessment scores for clinical scenario meningitis global (80.0 vs 67.1, p<0.05), splenomegaly raw (90.0 vs 74.3, p=0.01), global (84.3 vs 64.3, p<0.05) and combined (87.1 vs 69.3, p=0.01), and osteoporosis global (81.4 vs 60.0, p<0.05) and combined scores (75.5 vs 59.0, p<0.05) (Figure 1). The peer assessment scores were not less than faculty assessment scores in any post-graduate year or clinical scenario. Faculty and peer assessment scores did not differ between post graduate year (data not shown, p>0.05 for all comparisons).

Peer and faculty raw scores were highly correlated, with intra-class correlation (ICC) of 0.73 (95% confidence interval (CI) or 0.50-0.87). Peer and faculty global scores were not well correlated (ICC = 0.39).

## Discussion

The advent of CBME by RCPSC-certified internal medicine programs has made physical examination skills as EPAs. How to teach physical examination skills effectively remains a challenge with a number of techniques potentially showing promise: structured curriculum,^8^ multimedia-assisted teaching,^9^ simulation training,^10^ feedback,^11^ and instructor variation.^12-14^ Considerable concerns persist that adoption of CBME will increase demands for faculty time,^16-18^ and thus physical curriculum, to be compatible with CBME in a sustainable fashion, would benefit from decreasing time demands on faculty. This study evaluated the feasibility of a novel pilot curriculum designed to decrease burden on faculty.

This study showed a strong correlation between peer and faculty assessments for the raw, but not global scores. The raw score markings were based upon the combination of an evidence-based physical examination checklist and questions that assessed critical thinking and knowledge application, whereas the 10-point Likert scale was a subjective global rating of the candidate. Considerable bias has been reported in peer assessment in both medical and non-medical settings, including halo, horns, leniency, strictness, and similar-to-me biases.^13,22,23^ Within the medical education literature, the halo and friendship marking effects inflate peer assessment scores by peers compared to faculty,^24-26^ and inflated peer-assessment scores may be the norm in high stakes settings such as medical schools.^24^ The magnitude of peer assessment scores’ inflation was greater in the subjective global (13.0 points), than the more objective raw (7.0 points) scores. This was likely because of a reduction of the halo and friendship marking effects. On the other hand, the view of the faculty assessor into the formative OSCE room was farther than, and intermittently blocked by the peer assessor, so it is possible that this impaired the faculty assessor’s viewpoint to provide an accurate assessment of physical examination maneuvers. However, none of the four faculty members in this trial thought the view was ever hindered sufficiently to impair faculty assessment.

Medical literature confirms correlation between faculty and peer assessment may be low,^27,28^ medium,^26,29-32^ or high.^33^ The extent of correlation is largely predicted by the effect of biases in the assessment tool. In this study, peer raw scores overestimated faculty raw scores to less of an extent than global scores, and peer raw scores (but not global scores) strongly correlated with the faculty scores. Thus, implementation of an evidence-based physical examination checklist may neutralize common biases associated with peer assessment. Therefore, wider adoption of this curriculum and subsequent physical examination assessment should be based upon raw, rather than global, scores.

This study confirms that physical examination skills can be learned in the novel curriculum in a sustainable fashion with improved performance on a formative OSCE for those trainees who had previously been taught the physical examination topic. There are a number of reasons why trainees’ performance may have improved. Firstly, trainees created the physical examination evidence based checklist. This was done to decrease the workload for faculty physicians, but this act may in itself enhance knowledge retention.^34^ Secondly, the pilot curriculum sessions included both single trainee demonstration with faculty, and multiple peers practicing, a combination that has been shown to enhance physical examination skills.^13^ Thirdly, physical examination teaching by persons other than faculty physicians may be equally or more effective.^35-37^ Consequently, adoption of this curriculum should include all components so both intended and unintended benefits are mobilized. It is plausible that additional improvements in physical examination skills may be realized by supplementing this curriculum with other interventions such as physical examination videos: this remains a topic for ongoing research.

It is well established that learners tend to overestimate their skills, in self-assessment, yet the extent of this overestimation decreases with experience.^38-40^ The same is also true for peer assessment, which overestimated faculty score by 7.0 (peer-raw), 13.0 (peer-global) and 10.5 (peer-combined) scores in this study. Peer assessment tends to approximate faculty assessment scores as peer assessors become more experienced.^41,42^ This has important implications for curriculum design; peer assessment alone could lead to false confirmation of physical examination competency. Two potential solutions may address this. Firstly, a correction factor can be derived to adjust the peer-raw score to approximate the faculty-raw score. However, this will require intermittent faculty assessment to validate the correction factor prospectively. The alternative solution is to hold frequent faculty assessment without peer assessment. The first option may be more feasible if faculty time is in short supply as is the reality in many academic institutions. However, the required frequency of intermittent validation would warrant further study.

There are a number of important strengths in this study. Firstly, this is a strong report of a physical examination curriculum that can be adopted within the CBME framework that leads to sustainable improvements in trainee physical examination skills. Secondly, this study suggests that many of the biases of peer assessment may be diminished by use of raw scores from an evidence-based checklist, rather than subjective global scores. Thirdly, the curriculum could be generalized to other academic centers within the CBME framework, without substantial impairment of academic resources, including faculty time. In terms of weaknesses, firstly, this is a single center study consisting of one internal medicine program’s trainees. However, the Queen’s Internal Medicine program includes residents with diverse cultural backgrounds and subspecialty interests, and, thus, the results are likely generalizable to other large internal medicine programs. Secondly, the number of formative OSCE scenarios that were evaluated by both peers and faculty was low. On the other hand, the conclusions found in this study were both meaningful, normally distributed and statistically significant, and thus this did not decrease the importance of the study. Thirdly, this study was unable to determine which of the components of the curriculum led to the improvements in physical examination skills. However, the curriculum was designed to combine multiple educational methods, (self-directed learning, small group learning, and faculty led demonstration) while maintaining long-term sustainability within restricted faculty and department resources. Thus, the determination of which specific component is responsible is not as important as knowing that the entire curriculum can be replicated and delivered in other internal medicine training programs.

### Conclusion

This study confirms that peer assessments using raw scores, based on an evidence-based checklist, correlate well to faculty assessments, leaving an allowance for overestimation. A physical examination curriculum using checklist and demonstrations is sustainable in the CBME framework, with minimal faculty time commitment, leading to sustainable improvements in physical examination skills. Further research is required to determine how other co-operative peer run educational interventions could improve physical examination skills even further in this setting.

## Appendix A Example Checklist Prepared Prior to Practice Physical Exam Session

Examine the Patient to Differentiate Pleural Effusion and Pneumonia

- The task at hand is to distinguish the two entities via physical examination. Hence, only perform the maneuvers that have discriminatory values.

Basics

Introduce yourself to the patient

Wash hands

Expose and drape the patient as per appropriate

Respiratory Examination

Palpation

Tracheal Position: an effusion may push the tracheal contralaterally, and a consolidation may pull the trachea ipsilaterally.

Tactile Fremitus

e.g. Palpation of low frequency vibrations transmitted by voice through the chest *NB: Use sounds such as ‘oy’ (as in ‘boy’ or ‘toy’) as it better transmits low-pitched vibrations*

Percussion

Conventional Percussion

Auscultatory Percussion

Technique:
Have patient sit upright for 5 minutes
Place the diaphragm of the stethoscope on the posterior chest wall in midscapular line, approximately 3 cm below the last rib.
Use dominant hand to flick finger along three or more parallel vertical lines from apex towards base along each hemithorax
In pleural effusion: sharp change to loud percussion note at superior edge of pleural effusion

Auscultation

Breath Sounds (e.g. bronchial breath sounds, diminished breath sounds)
Adventitious Sounds (e.g. crackles - nonmusical, discontinuous lung sounds; rhonchi - rattling, continuous low-pitched lung sounds)
Pleural Rub
Vocal Fremitus
Egophony (change in timbre of vowel sounds from ‘e’ to ‘a’)
Whispered Pectoriloquay (increased clarity of whispered phrases)

**Table 1:** Physical Exam Findings Differentiating Pleural Effusions versus Pneumonia

Physical Finding	Pleural Effusion	Pneumonia
Tracheal Deviation	Deviate Contralaterally	Deviate Ipsilaterally
Tactile Fremitus	Reduced	Increased
Percussion	Reduced (‘stony dullness’)	Reduced
Breath Sounds	Reduced	Bronchial
Adventitious Sounds	Pleural Friction Rub (if minimal fluid with pleurisy) Crackles (heard superior to effusion)	Coarse Crackles Rhonchi
Special Tests		Vocal Fremitus (e.g. egophony, bronchophony, whispered pectoriloquy)

Accuracy of Physical Exam Maneuvres:

What is the best test to rule in pleural effusion?
Dullness to conventional percussion [positive LR +8.7, 95% CI 2.2-33.8]
What is the best test to rule out pleural effusion?
Absence of reduced tactile vocal fremitus [negative LR -0.21, 95% CI, 0.12-0.37]
What is the best test to rule in/out pneumonia?
Trick question! No single physical exam maneuvre is sufficient to rule in/out pneumonia.
Combination of asymmetrical chest expansion [positive LR ∝, 95% CI 3.2-∝], egophony or dullness to percussion increased the likelihood of pneumonia
Absence of three vital sign abnormalities was found to have a low negative LR (e.g. RR <30 bpm, HR <100 bpm, T <37.8) [negative LR -0.18, 95% CI 0.07-0.46].
If considering pneumonia, radiographic imaging is required in addition to history and physical exam to reliably rule in/out pneumonia.

**Sources used in the creation of this document:**

- Metlay JP, Kapoor WN, Fine MJ. Does this patient have community-acquired pneumonia? Diagnosing pneumonia by history and physical examination. *JAMA*. 1997;278(17):1440-5.
- Wong CL, Holroyd-Leduc J, Straus SE. Does this patient have a pleural effusion? *JAMA*. 2009;301(3):309-17.

## Appendix B Marking Scheme for OSCE Station

**(includes Candidate instructions and Examiner Marking Sheet)**

### INSTRUCTIONS to CANDIDATE

You are the internist in a community hospital. You are about to see Mr David Raynold a 23 year old college student, who is complaining of headache and fever for 3 days. He denies any altered level of consciousnesses or seizure activity. He denies any travel or contact history. His past medical history is significant for migraine for which he takes Advil PRN. He is a non smoker and does not consume any alcohol.

You will have **six minutes** to do the following:

A) **What is your leading diagnosis?**
B) **You will be asked to examine this patient for your leading diagnosis**
C) **What investigation/s would you order to confirm your diagnosis**

**More questions will follow after 6 minutes**

### EXAMINER SHEET

**Examiner Name: ____________________________________**

**Candidate Name: ____________________________________**

**Candidate Training Level (circle one):**

**PGY-1 PGY-2 PGY-3**

**Instructions to Examiner: Candidate has 6 minutes to do A, B and C, then examiner should move on to D.**

**A) Ask candidate: “What is your leading diagnosis?”**
  Correct answer = Meningitis ***(one mark for correct answer)***
B) **Ask candidate: “Please examine this patient for your leading diagnosis”**

**General** ***(one checkmark for each)***

- Introduce self and wash hands
- Vital signs: (?Fever? Shock?), Chills, rigors

**Assessing for Meningeal Inflammation** ***(one checkmark for each)***

**MUST PERFORM**

1. Nuchal rigidity: +ve if resistance during gentle forward flexion while patient supine
2. Kernig: +ve if resistance or pain in the lower back or posterior thigh hip during extension of knee in supine patient with hips and knees flexed at 90 degrees
3. Brudzinski: +ve if flexion knees/hips during passive neck flexion in supine patient
4. Jolt accentuation of headache: + if worsening of baseline HA while patient actively turns head horizontally at frequency of 2-3 rotations per second
5. Photophobia

**Extracranial findings – looking for etiology**

**MUST MENTION without prompting, but need not perform**
**(one checkmark if gets 2 of 3 in list)**

1A. Rash (Vesicular lesions VZV, genital vesicles HSV-2, Petechial)
1B. Head and Neck (Otoscopy for otitis media, Rhinoscopy for purulent Lx, Pharyngeal exam for purulence/erythema, Lymphadenopathy)
1C. Respiratory exam: assess for presence of pneumonia

**Neurologic Exam – looking for complications**

**MUST MENTION without prompting, but need not perform**
Signs of increased ICP: (Hypertension, bradycardia, altered LOC, seizures, nausea/vomiting, fundoscopy for papilloedema)
  ***(one checkmark for signs of increased ICP)***
Mental status exam
  - Cranial nerve exam (especially palsies of CN 3, 4, 6, 7)
  - Motor and sensory, Cerebellar testing, Reflexes (including Babinski)
  - **(one checkmark for two of the three above)**
  - **Physical Exam sensitivity, specificity and likelihood ratios**

**Table:**

	Sens (%)	Spec (%)	LR +	LR -
**Jolt Accentuation** (based on 1 study)	97	54	2.4	0.05
**Fever**	43	48	0.82	1.2
**Neck Stiffness**	3-15	68	0.94-6.6	0.83-1.0
**Brudzinski**	5	95	0.97	1.0
**Kernig**	5-9	95	0.97-4.2	0.92-1.0
***Altered mental status***	*69*	*-*	*-*	*-*
***Focal neuro findings***	*21*	*-*	*-*	*-*
***Rash***	*61*	*-*	*-*	*-*

Consolidated from JAMA RCE + Update

C) **What investigation/s would you order to confirm diagnosis?**
  CBC, Lactate, Blood cultures **(one mark for any two)**
  Lumbar puncture for cell count, differential, culture/gram stain, glucose, protein **(one mark for all)**
  May be required: Liver function tests (INR, PTT, AST, ALT, Total Bilirubin, Albumin), Urine culture, CXR, Lytes, Cr ***(one mark if 3 of the above are mentioned)***
D) **At six minutes, MOVE ON and say to candidate**
  “You decide to perform a lumbar puncture on this patient. Please counsel this patient on this procedure. You do NOT have to explain the procedure to the patient.”
  **DO NOT PROMPT CANDIDATE**
   Indications ***(one mark):***
  Rule out life threatening infection, SAH or disabling brain disorders
   Benefits ***(one mark):***
  - Can confirm or exclude and guide management Risks: Undiagnostic result, failure to obtain specimen, need for repeat
   Common: ***(one mark)***
  - - **Post procedure HA** – often occurs within 72h or procedure and lasts up to 5d
    - Procedure site backache – usually last several days
  - Uncommon
    - Paresthesias: shooting pain down legs until needle out
  - Rare ***(one mark):***
    - Infection introduced by LP
    - **Spinal hematomas** – especially in coagulation abnormalities.
    - **Brain herniation/coning** (most severe, but extremely rare in neurologically normal patient) – leading to death or disability
   Alternatives ***(one mark)***
  - MRI can assess radiologic changes, but not as good to assess actual CSF composition Not doing (implications) ***(one mark)***
  - Not receiving the correct treatment for the appropriate organism
  - Receiving antimicrobial treatment incorrectly
  Summarize (ask for any questions)
  **Global** performance (1 = needs significant improvement, 10 = outstanding)
  **Table:**
  1	2	3	4	5	6	7	8	9	10
  **Did the candidate participate in the group practice**
  **(Wednesday afternoons) for this particular exam station? (circle one)**
  **Yes No**
